# TNFAIP8 family gene expressions in the mouse tail intervertebral disc injury model

**DOI:** 10.1002/jsp2.1093

**Published:** 2020-06-19

**Authors:** Zuozhen Tian, Frances S. Shofer, Lutian Yao, Honghong Sun, Hongtao Zhang, Ling Qin, Youhai H. Chen, Yejia Zhang

**Affiliations:** ^1^ Department of Physical Medicine & Rehabilitation University of Pennsylvania Philadelphia Pennsylvania USA; ^2^ Department of Emergency Medicine University of Pennsylvania Philadelphia Pennsylvania USA; ^3^ Department of Orthopaedic Surgery University of Pennsylvania Philadelphia Pennsylvania USA; ^4^ Department of Orthopaedics/Sports Medicine and Joint Surgery, First Affiliated Hospital China Medical University Shenyang Liaoning China; ^5^ Pathology and Laboratory Medicine, Perelman School of Medicine University of Pennsylvania Philadelphia Pennsylvania USA; ^6^ Translational Musculoskeletal Research Center (TMRC) Corporal Michael J. Crescenz Veterans Affairs Medical Center Philadelphia Pennsylvania USA

**Keywords:** immune response, inflammation, injury

## Abstract

**Introduction:**

The TNF‐α‐induced protein‐8 (TNFAIP8, also known as TIPE) family of molecules comprises four members: TNFAIP8 and TIPEs1‐3. Since the first description of these proteins, their roles in fine‐tuning inflammation and in directing leukocyte migration have been described in several organ systems. However, their relationship with intervertebral disc (IVD) is unknown.

**Materials and methods:**

Here, we describe the expression of TNFAIP8 family genes in the nucleus pulposus (NP) and annulus fibrosus (AF) of the normal adult murine IVD. We further describe the expression of these genes in the injured male and female murine IVD.

**Results:**

*Tnfaip8* gene expression was decreased, and *Tipe1* gene expression was essentially unchanged, in response to injury. *Tipe2* and *Tipe3* gene expression was markedly elevated in response to IVD injury, along with those encoding known inflammatory markers (ie, *Tnfa, Il6, Cxcl1*, and *Adam8*). Additionally, sex‐related differences were also observed for some of these genes in intact and injured mouse IVDs. Future studies include examining tissue distribution of TNFAIP8 family proteins and identifying cells that produce them. In addition, examining mice that are deficient in TNFAIP8 molecules, in relation to gene expression, tissue morphology and mouse behavior, may further delineate the roles of these molecules in IVD inflammation and degeneration.

## Graphical abstract

The TNFα‐induced protein‐8 (TNFAIP8, also known as TIPE) family of molecules comprises four members: TNFAIP8 and TIPEs1‐3. Here, we describe the expression of these genes in the injured murine intervertebral discs. *Tnfaip8* gene expression was decreased, and *Tipe1* gene expression was essentially unchanged, in response to injury. *Tipe2* and *Tipe3* gene expression was markedly elevated in response to disc injury, along with those encoding known inflammatory markers (i.e., *Tnfa*, *Il6*, *Cxcl1*, and *Adam8*).

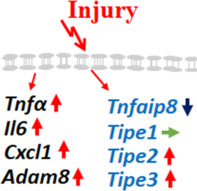

## 1 INTRODUCTION

Back pain related to intervertebral disc (IVD) degeneration costs billions of dollars in the U.S.^1^, ^2^ Current treatments include surgical and nonsurgical approaches, and often result in incomplete symptom relief because the molecular relationships between back pain and IVD degeneration are still unclear. Clinically, patients with chronic back pain often have histories of injuries. Uncovering mechanisms of disc inflammation following injury may shed light on the mechanism of spinal pain.

The TNFAIP8 (tumor necrosis factor‐α‐induced protein 8; TNFAIP8) family are newly described regulators of immunity and tumorigenesis consisting of four highly homologous mammalian proteins: TNFAIP8, TIPE1 (TNFAIP8‐like 1, or TNFAIP8L1), TIPE2 (TNFAIP8L2), and TIPE3 (TNFAIP8L3). They are novel signaling proteins recently described to be key factors regulating inflammation and oncogenesis.^3^, ^4^, ^5^ Interestingly, despite significant sequence homology among the four members of this family,^5^ they are involved in different biological activities and exhibit remarkable variability of expression.^6^ Furthermore, this family of proteins is highly dysregulated in arthritis, cancers and various other chronic diseases.^6^

TNFAIP8 is a founding member of the TNFAIP8 family, originally identified in head and neck cancer cells,^7^ and in cells treated with TNF‐α.^8^ TNFAIP8 is a negative regulator of apoptosis, and is oncogenic.^4^, ^5^ Moreover, TNFAIP8 protein inhibits apoptosis by inhibiting Caspase‐8.^9^ Knocking out the *Tnfaip8* gene exacerbated disease in a dextran sodium sulfate (DSS) model of murine colitis.^10^ Indeed, phospho‐AKT (also known as protein kinase B [PKB]) activity decreased in TNFAIP8 knockout colonic cells, indicating that TNFAIP8 may regulate epithelial cell death by targeting AKT.^11^ The increased mortality in TNFAIP8 knockout mice with colitis could be explained by increased cell death, and decreased proliferation of colonic epithelial cells.^11^

In mice, TIPE1 was found to be expressed in a wide variety of tissues and cells, including neurons, hepatocytes, muscle tissue, intestinal epithelial cells, and germ cells. TIPE1 interacts with Rac1 and inhibits activation of downstream p65 and c‐Jun N‐terminal kinase signaling, which increased caspase‐mediated apoptosis.^12^

TIPE2 was discovered by genomic profiling of inflamed neural tissues.^10^, ^13^ TIPE2 was found to be widely expressed in lymphoid tissues; its knockout in mice resulted in the development of spontaneous multiorgan inflammation, splenomegaly and premature death, and these mice were hypersensitive to toll‐like receptor (TLR) stimulation.^10^ Recently, it was found that leukocyte polarity was generated by TIPE2, a transfer protein for phosphoinositide second messengers. TIPE2 functioned as a local enhancer of phosphoinositide‐dependent signaling and cytoskeleton remodeling, which promoted leading‐edge formation.^14^

TIPE3 is the most recently investigated TNFAIP8 family member. TIPE3 protein is preferentially expressed in reproductive and neural tissues, with nearly identical murine and human expression profiles.^15^ TIPE3 is the transfer protein of lipid second messengers that promote cancer.^16^

Several TIPE proteins have been crystallized.^17^, ^18^ Knockout mice for TNFAIP8 and TIPE2 have also been generated,^10^, ^11^ and should be valuable for studying inflammation in the musculoskeletal system, including back pain related to IVD injuries. Inflammatory cytokines and chemokines have been found in painful/degenerative human IVDs.^19^, ^20^, ^21^ However, TNFAIP8 family gene expression in mouse IVDs has not been described previously. This study is the first that describes gene expression profiles of the TIPE molecules in response to injury, an important step toward understanding the roles of the TNFAIP8 family of proteins in IVD inflammation.

The mouse model permits examination of IVD degeneration in mice with genetic modifications, allowing the role of a specific molecule in the disease process to be studied. Overall, mice and humans share virtually the same set of genes. Almost every gene found in one species so far has been found in a closely related form in the other. It is, however, important to be aware of the limitations of modeling human spinal disease using animals,^22^ and to recognize that the biomechanical properties of the mouse tail differ from those of the human spine. The mouse tail IVD injury model was established by Yang et al^23^ and Martin et al.^24^ We have recently refined the procedure using a percutaneous needle puncture approach, and have shown that needle injury results in a reproducible course of morphological and molecular changes consistent with IVD degeneration.^25^, ^26^ Among the four time points we studied,^26^ reproducible changes in gene expression and histology occurred at 1 week post injury. Accordingly, this time point was selected to examine TNFAIP8 family member gene expression.

DBA mice are used in the musculoskeletal field, because they are more susceptible to some types of arthritis than the C57BL/6 (also known as B6) strain.^27^ We have found that ADAM8 plays important roles in arthritis^28^, ^29^, ^30^ and in IVD degeneration.^31^ Our ongoing work on the role of Adam8 in intervertebral disc degeneration uses a mouse with ADAM8 inactivated by introducing the E330Q mutation into its proteolytic domain.^30^ This animal is on the DBA background, and the *Tnfaip8* gene expression studies are performed with these mice.

Since TNFα is a known inducer of the TNFAIP8 family, the *Tnfα* gene was also included in the panel of genes examined. We have also examined genes encoding inflammatory cytokines/chemokines related disc degeneration such as *Il6* ^20^ and *Cxcl1* ^21^ in this study. The *Adam8* gene was included because of its relationship with inflammation and fibronectin cleavage in the IVD.^31^

Finally, in order to comment on potential differences in gene expression pattern between male and female animals per National Institutes of Health (NIH) guidelines, we have examined previously mice of both sexes and found subtle but at times significant differences in expression patterns of selected genes.^32^ Therefore, we have analyzed data from male and female mice separately in the present study.

## 2 MATERIALS AND METHODS

### 2.1 Mice

All animal experimental procedures were approved by the Institutional Animal Care and Use Committee of the University of Pennsylvania, Philadelphia, PA. Thirty young adult mice 10 to 11 weeks of age, on the DBA background (DBA/1LacJ, the Jackson Laboratory, Bar Harbor, Maine) were used in this study. Twelve female and 12 male mice were used for tail IVD injury experiment, and 6 female mice were used to isolate nucleus pulposus (NP) and annulus fibrosus (AF) tissues for RNA extraction. Mice were housed under pathogen‐free conditions with environmental enrichment (nestlets by Ancare, Bellmore, New York), and up to five animals per cage. Mice were fed PicoLab diet no. 5053 (LabDiet, Fort Worth, Texas) without restriction, provided with acidified bottled water, and maintained on a 12:12‐hours light:dark cycle. Room temperature is at 21.1°C to 24.4°C (equivalent to 70 F‐76 F) and humidity at 30% to 70%.

### 2.2 NP and AF dissection

The lumbar and coccygeal vertebrae were isolated with a scalpel under a dissecting microscope (VistaVision, VWR International, Radnor, Pennsylvania), as described previously.^33^ The gelatinous NP was scraped off with a scalpel. AF tissues, identified by their concentric rings, were shaved off the cartilaginous endplate with a scalpel. Lumbar and coccygeal IVDs were pooled for each animal.

### 2.3 Tail injury surgery

Surgery was performed as described previously.^26^ Specifically, each mouse was anesthetized with Ketamine (90 mg/kg) and Xylazine (10 mg/kg) subcutaneously. Under anesthesia, the skin was cleaned with betadine. Under fluoroscopic guidance with a mini C‐arm (OrthoScan FD Pulse Mini C‐Arm, Orthoscan Inc., Scottsdale, AZ), the mouse coccygeal (Co) IVDs were identified, and a 26G needle was inserted into the IVD space until the needle tip reached ~2/3 of the disc thickness. This information has been included in our recent manuscript as a supplemental figure.^26^ Care was taken not to puncture the opposing wall of the AF. Indeed, when the opposing AF wall was damaged, a more severe injury was seen on MRI.^25^ Gelatinous tissues were often found on the needle tip after this had been removed, suggesting that the needle puncture induces an acute herniation of the gelatinous NP of the IVD. In the current study, the Co3/4 and Co5/6 IVDs in each mouse were injured, while Co4/5 and Co6/7 served as intact controls, as described previously.^26^ Mice were checked 4 hours after surgery and the next day. The mice were then monitored daily until the endpoint. Even though mice did not show any signs of pain or distress, as a pre‐emptive treatment, mice were given buprenorphine at the time of sedation and 4 hours later. Animals were sacrificed by exposure to CO_2_ at 1 week after tail disc injury. From each mouse tail, Co3/4 (injured) and Co4/5 (intact control) discs were isolated individually for RNA extraction. Specifically, IVD tissues were separated from their adjacent cartilaginous endplates and bone with a scalpel, under a dissecting microscope (VistaVision, VWR International, Radnor, Pennsylvania). Co5/6 (injured) and Co6/7 (intact control) were isolated en bloc for histological examination.

### 2.4 RNA isolation and quantitative real‐time PCR

Total cellular RNA was isolated by the Trizol method as described previously.^26^, ^33^ Specifically, the isolated IVD tissues were soaked in RNALater (Ambion, Foster City, California) overnight, and stored at −80°C until extraction. On the day of RNA extraction, RNALater was removed and the tissues were snap frozen with liquid Nitrogen, pulverized, and then transferred into Trizol (Invitrogen, Carlsbad, California). The tissues were homogenized with a homogenizer with disposable OmniTip probes for hard tissue (Omni International, Kennesaw, Georgia). RNA was precipitated with 70% ethanol, and was further purified using a RNeasy Micro Kit (Qiagen). RNA concentration was determined using a Synergy H4 Hybrid Reader (BioTek, Winooski, Vermont). To generate cDNA, all RNA from each IVD was used as template in a reverse transcriptase reaction using the SuperScript VILO cDNA synthesis kit (Life Technologies, Carlsbad, California) containing random hexamers, and added polyDT primers (Invitrogen, Carlsbad, California). CDNA sequences were retrieved from Ensembl (ensembl.org, release 84, March 2016). Primers for real‐time PCR (*Gapdh*, *Tnfa*, *Il6, Cxcl1*, and *Adam8* genes) were designed using Primer‐BLAST,^34^ and synthesized by Invitrogen (Carlsbad, California), as described previously.^26^ Primers for *Tnfaip8, Tipe 1‐3* were purchased (QuantiTect Primer, Qiagen). For each PCR reaction, cDNA, SYBR Select master mix (Life Technologies, Carlsbad, California), and primers (working concentration 0.5 μM) were mixed, and deionized water was added to a total volume of 20 μL per reaction. MicroAmp Optical 96‐well reaction plates (Applied Biosystems, Foster City, California) with 20 μL of reaction mix/well were sealed with optical adhesive film (Life Technologies, Frederick, Maryland) and run in a ViiA7 real‐time PCR system (Applied Biosystems, Foster City, California) using the following program: (1) 50°C for 2 minutes, (2) 95°C for 2 minutes, (3) 95°C for 15 seconds, (4) 58°C for 1 minute, (5) repeat steps 3 and 4 a total of 40 times. Single products were confirmed by determining melting curves at the conclusion of the reaction. Expression of each gene was normalized to gapdh (reference gene), and results are expressed as target gene/gapdh ratio, equal to 2^‐ΔCt^.^35^, ^36^

### 2.5 Histological evaluation

The IVDs and portions of the adjacent bony vertebral bodies were isolated immediately after euthanasia. The disc with its surrounding vertebral bodies was fixed with 4% paraformaldehyde for 24 hours. The bone‐disc‐bone segments were decalcified with a solution consisting of 12.5% EDTA for ~1 week, with shaking, until the bony portion was completely decalcified.^26^ The tissues were then dehydrated and embedded in paraffin and sectioned to 5 μM thickness. For Picrosirius red staining, sections were stained with 0.1% Picrosirius red (Sigma) for 45 minutes. All samples were examined under a light microscope (Nikon) and photographed.

### 2.6 Statistics

The expression ratio for genes of interest (*Tnfaip8, Tipe1‐3, Il6, Cxcl1, Adam8*, and *Tnfα*) and house‐keeping gene (*Gapdh)* were calculated for each sample. If expression ratio was not normally distributed, data were log transformed. To assess differences in gene expression ratio, two‐way analysis of variance (ANOVA) in repeated measures where the grouping factor was sex, and the repeated measure injury/intact were performed. To minimize type I error, post hoc pairwise comparisons using Tukey‐Kramer tests were performed to examine differences between injured/intact tissues and tissues from mice of different sexes. A *P*‐value of <.05 was considered statistically significant. All analyses were performed using SAS statistical software (Version 9.4, SAS Institute, Cary, North Carolina).^37^

## 3 RESULTS

### 3.1 *Tnfaip8* family gene expression in the intact NP and AF tissues

To examine whether *Tnfaip8* family of genes is expressed in the intact mouse IVDs, we used mice that have not had tail IVD injuries. We isolated NP and AF tissues as described previously.^33^ The expression ratio for genes of interest (*Tnfaip8*, and *Tipe1‐3*) and housekeeping gene (*Gapdh*) were calculated for NP and AF. There was detectable expression of all four members of the TNFAIP8 family, although the overall levels were low compared with those of the extracellular matrix molecules.^33^

**FIGURE 1:**
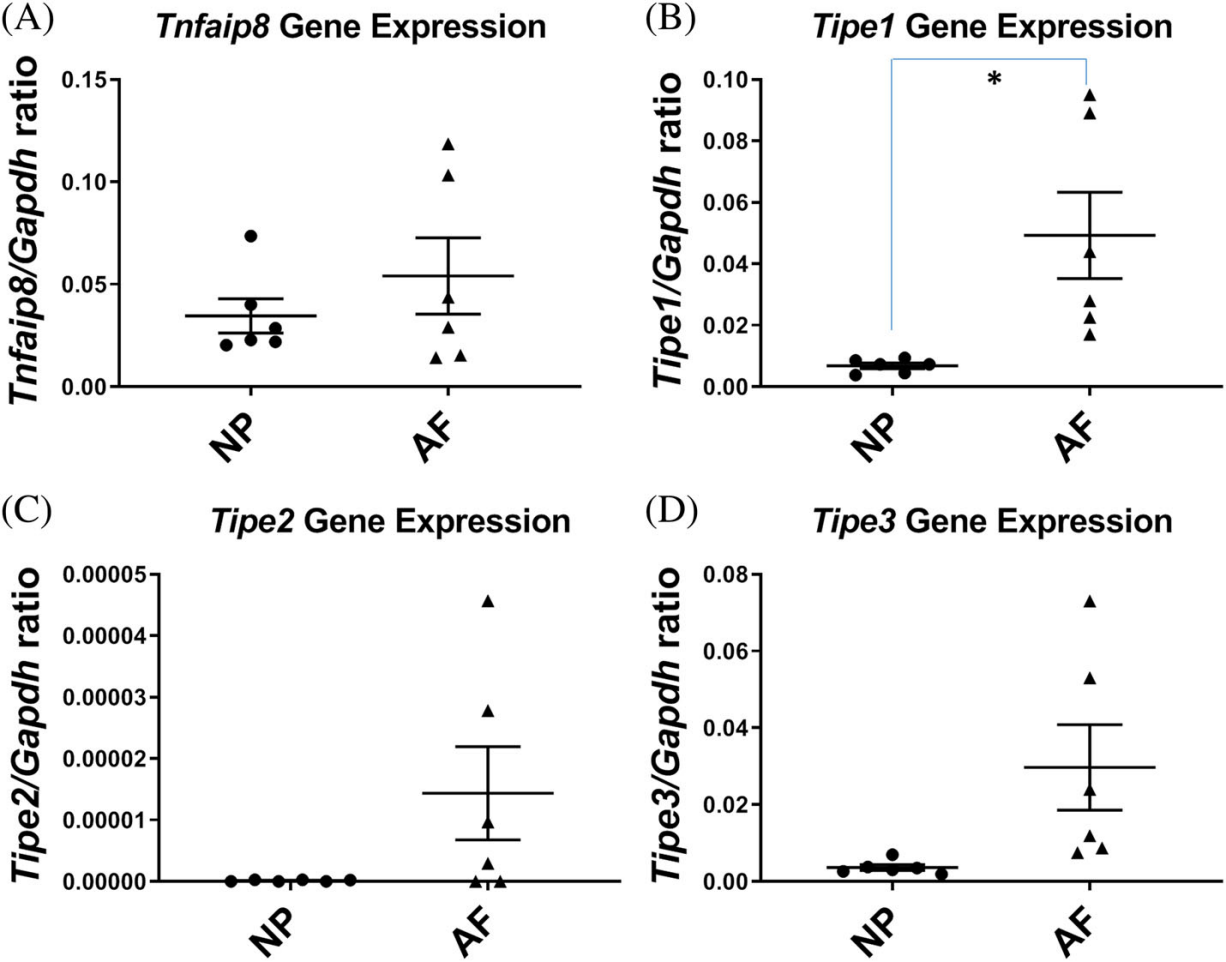
*Tnfaip8* family gene expression in the nucleus pulposus (NP) and annulus fibrosus (AF) of the intact mouse tail intervertebral disc. A, *Tnfaip8* gene expression; B, *Tipe1* gene expression; C, *Tipe2* gene expression; D, *Tipe3* gene expression. Each point shows data from one intervertebral disc. *P ≤ .05

There was no statistically significant difference in *tnfaip8* gene expression between NP and AF *(Tnfaip8* to *Gapdh* gene expression ratio: 0.0346 and 0.0540 in the NP and AF, respectively; n = 6 mice, *P* = .2049; Figure 1A). *Tipe1* was expressed at a higher level in the AF than in the NP (*Tipe1* to *Gapdh* ratio 0.0068 and 0.0493 in the NP and AF, respectively; n = 6; *P* = .0312; Figure 1B). The gene expression level of *Tipe2* was very low in both NP and AF tissues (*Tipe2* to *Gapdh* ratio: 1.27 × 10^−7^ and 1.44 × 10^−5^ in the NP and AF, respectively; n = 6, *P* = .1181; Figure 1C). It is worth mentioning that these levels are very low and quantification may not be reliable with the real‐time PCR methods used here. *Tipe3* gene showed a tendency to higher expression in the AF than in the NP (*Tipe3* to *Gapdh* ratio 0.0036 and 0.0297 in the NP and AF, respectively; n = 6; *P* = .0582; Figure 1D). Among the four genes, *Tnfaip8* gene expression is the highest, and *Tipe2* gene expression lowest both in NP and AF, with *Tnfaip8* gene expression 5 and 3 orders of magnitude of *Tipe2* in NP and AF, respectively (n = 6).

### 3.2 *Tnfaip8* family gene expression in the injured tail IVDs

**FIGURE 2:**
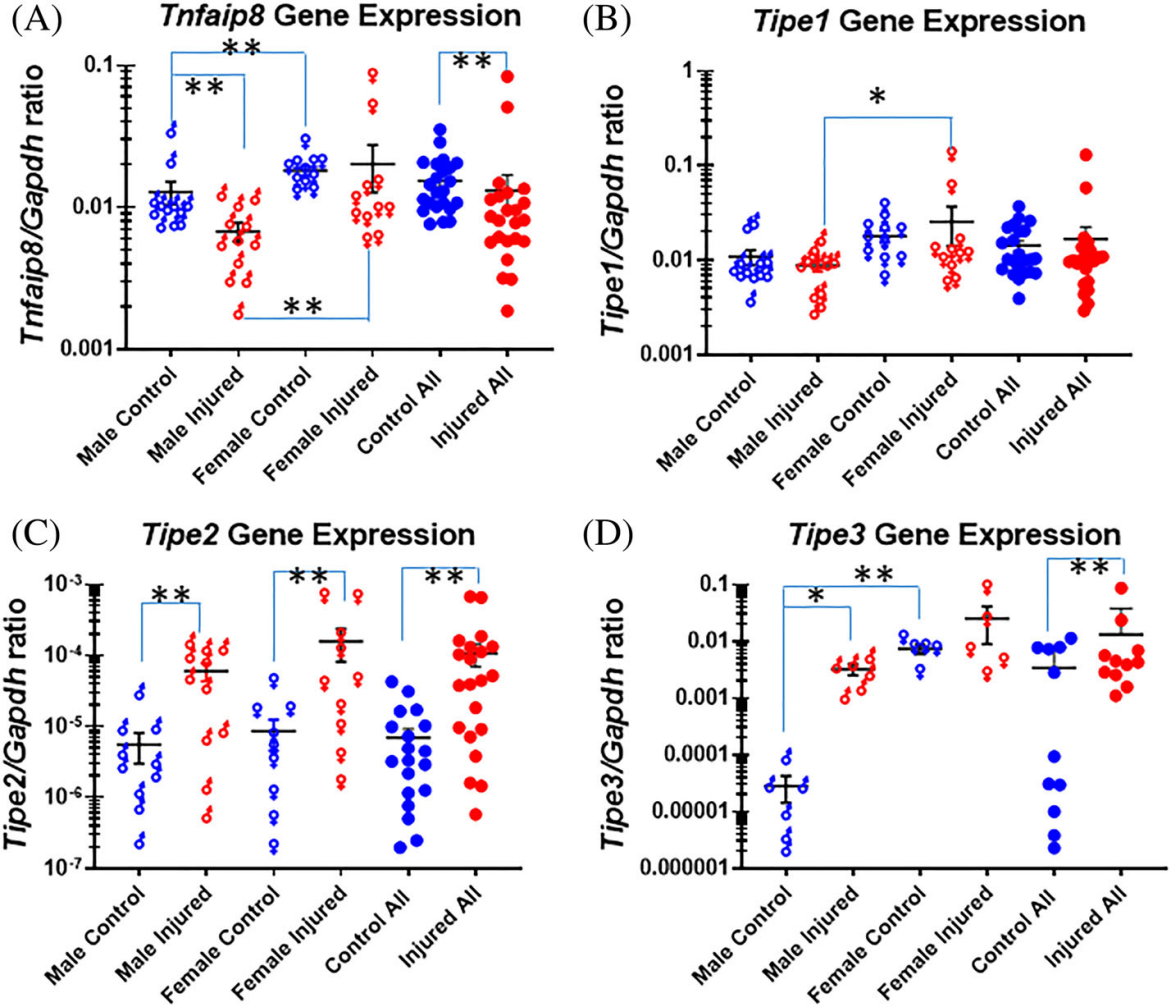
Gene expression of *Tnfaip8* family in the injured mouse tail intervertebral disc. A, *Tnfaip8* gene expression; B, *Tipe1* gene expression; C, *Tipe2* gene expression; D, *Tipe3* gene expression. Each point shows data from one intervertebral disc. **P ≤ .01; *P ≤ .05

In male mice, *Tnfaip8* gene expression decreased in response to injury, compared with intact controls (*Tnfaip8* to *Gapdh* gene expression ratio: 0.0068 in injured IVDs and 0.0128 in controls respectively; n = 12 mice; *P* = .0017; Figure 2A). In female mice, however, there was no statistically significant difference in *Tnfaip8* gene expression in injured and intact IVDs (*Tnfaip8* to *Gapdh* gene expression ratio: 0.0215 in injured, 0.0181 in controls, respectively; n = 11 mice; *P* = .1547). When analyzing all male and female mice together, *Tnfaip8* gene expression decreased in injured discs compared with intact controls (n = 23, *P* = .0019; Figure 2A). *Tnfaip8* gene expression was higher in female controls than in male control mice (*P* = .0333). Similarly, *Tnfaip8* gene expression was higher in female injured than male injured discs (*P* = .0003; Figure 2A).

There was no statistically significant change in *Tipe1* gene expression in injured IVDs compared with that in intact controls either in male mice (*Tipe1* to *Gapdh* gene expression ratio: 0.0088 in injured, 0.0109 in controls respectively; n = 12 mice; *P* = .4525) or in female mice (*Tipe1* to *Gapdh* gene expression ratio: 0.0255 in injured, 0.0180 in controls respectively; n = 11 mice; *P* = .7232). When analyzing all male and female mice together, *Tipe1* gene expression in injured vs intact controls still did not show any statistical significance (n = 23, *P* = .2032). Similar to the *Tnfaip8* gene, *Tipe1* showed a trend for higher gene expression in female intact control discs than in male controls (*P* = .0599). The gene expression ratios in the injured IVDs are higher in injured females than males (*P* = .0274; Figure 2B).

*Tipe2* gene expression, on average, is three orders of magnitude lower in the intact IVDs compared with that of *Tnfaip8* (n = 23 mice). *Tipe2* gene expression increased significantly in mice of both sexes in response to injury, compared with intact controls. In male mice, *Tipe*2 to *Gapdh* gene expression ratios were 60.67 × 10^−6^ in injured discs and 5.55 × 10^−6^ in controls, respectively (n = 12 mice; *P* = .0048). In female mice, *Tipe2* gene expression also increased in injured compared with intact IVDs (*Tipe2* to *Gapdh* gene expression ratio: 159.41 × 10^−6^ in injured, 8.53 × 10^−6^ in intact controls, respectively; n = 11 mice; *P* = .0024). When analyzing all male and female mice together, *Tipe2* gene expression in injured IVDs was clearly elevated compared with that in intact IVDs (*Tipe2* to *Gapdh* gene expression ratio: 107.90 × 10^−6^ in injured IVDs and 6.97 × 10^−6^ in controls, respectively; n = 23, *P* = .0001). There was no statistically significant difference in *Tipe2* gene expression between male and female mice, in either injured or control discs (*P* > .0500; Figure 2C).

*Tipe3* gene expression increased significantly in male mice in response to injury, compared with intact controls (*Tipe3* to *Gapdh* gene expression ratio: 322 × 10^−5^ in injured IVDs and 2.84 × 10^−5^ in controls, respectively; n = 6 mice; *P* < .0001). In female mice, there was no statistically significant difference in *Tipe3* gene expression in injured vs intact controls (*Tipe3* to *Gapdh* gene expression ratio: 2497.85 × 10^−5^ in injured, 743.22 × 10^−5^ in intact controls, respectively; n = 5 mice; *P* = .7997). When analyzing all male and female mice together, *Tipe3* gene expression in injured IVDs increased significantly compared with that in intact IVDs (*Tipe3* to *Gapdh* gene expression ratio: 1312 × 10^−5^ in injured IVDs and 339 × 10^−5^ in controls, respectively; n = 11, *P* < .0001). This interesting pattern of change in response to injury is due, at least in part, to higher *Tipe3* gene expression in female control IVDs than in male controls (*P* < .0001; Figure 2D).

### 3.3 Proinflammatory cytokine (*Tnfα*, *Il6*, *Cxcl1*) and *Adam8* gene expression

**FIGURE 3:**
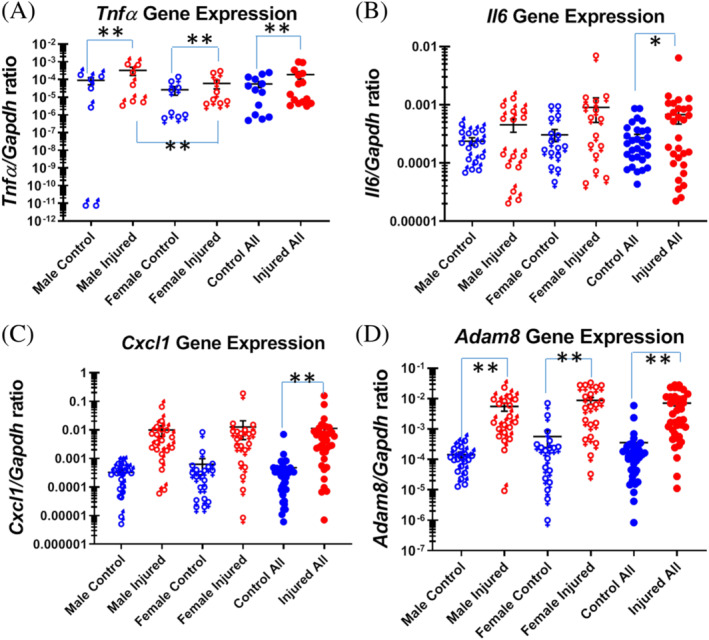
Pro‐inflammatory gene expression in the injured mouse tail intervertebral disc. A, *Tnfα* gene expression; B, *Il6* gene expression; C, *Cxcl1* gene expression; D, *Adam8* gene expression. Each point shows data from one intervertebral disc. **P ≤ .01; *P ≤ .05

*Tnfα* gene expression is elevated in the injured IVDs compared with that in intact controls in both male mice (*Tnfα* to *Gapdh* gene expression ratio: 33.45 × 10^−5^ in injured disc, 9.18 × 10^−5^ in controls; n = 7 mice; *P* = .0011) and female mice (*Tnfα* to *Gapdh* gene expression ratio: 6.07 × 10^−5^ in injured, 2.62 × 10^−5^ in controls respectively; n = 8 mice; *P* = .0009). When combining the data from both sexes, *Tnfα* gene expression is elevated (*Tnfα* to *Gapdh* gene expression ratio: 18.84 × 10^−5^ in injured discs, 5.63 × 10^−5^ in controls; n = 15 mice; *P* < .0001; Figure 3A). Surprisingly, the males expressed higher levels of *Tnfα* than did female mice, both in injured discs and controls (*P* = .0004 and *P* = .0005 for injured and control discs, respectively; Figure 3A).

The increase in *Il6* gene expression did not reach statistical significance in either male or female mice when comparing injured discs with intact controls (n = 15 mice/group; *P* =.9818 and .3085, respectively). When data from both male and female mice are analyzed together, *Il6* gene expression elevation in injured IVDs compared with controls reached statistical significance (*Il6* to *Gapdh* gene expression ratio: 6.79 × 10^−4^ in injured discs, 2.71 × 10^−4^ in controls; n = 30 mice; *P* = .0437). There was no statistically significant difference between the male and female mice, either in injured discs or controls (n = 15 mice/group; *P* > .0500; Figure 3B).

There was no statistically significant increase in *Cxcl1* gene expression in the injured compared with control discs in male mice (*Cxcl1* to *Gapdh* gene expression ratio: 98.87 × 10^−4^ in injured discs, 3.26 × 10^−4^ in controls; n = 19 mice; *P* = .3594). In female mice, there is a trend for increase in *Cxcl1* gene expression (*Cxcl1* to *Gapdh* gene expression ratio: 126.50 × 10^−4^ in injured, 6.25 × 10^−4^ in controls; n = 19 mice; *P* = .0748). When analyzing male and female mice together, the *Cxcl1* gene expression was significantly higher in the injured discs than in intact controls (*Cxcl1* to *Gapdh* gene expression ratio: 112.68 × 10^−4^ in injured, 4.76 × 10^−4^ in controls; n = 38 mice; *P* = .0055). There was no statistically significant difference between the male and female mice, either in injured discs or controls (n = 19 mice/group; *P* > .0500; Figure 3C).

The increase in *Adam8* gene expression in the injured compared with control discs in male mice was significant (*Adam8* to *Gapdh* gene expression ratio: 55.15 × 10^−4^ in injured, 1.44 × 10^−4^ in controls; n = 19 mice; *P* < .0001). In female mice, *adam8* gene expression was similarly elevated (*Adam8* to *Gapdh* gene expression ratio: 87.37 × 10^−4^ in injured discs, 5.67 × 10^−4^ in controls; n = 19 mice; *P* < .0001). When analyzing male and female mice together, the *Adam8* gene expression was higher in the injured discs than in intact controls (*Adam8* to *Gapdh* gene expression ratio: 71.26 × 10^−4^ in injured discs, 3.56 × 10^−4^ in controls; n = 38 mice; *P* < .0001; Figure 3D). There was no statistically significant difference between the male and female mice, either in injured discs or in controls (n = 15 mice/group; *P* > .0500; Figure 3D).

### 3.4 Tail IVDs showed consistent histological changes following injury in mice of both sexes

**FIGURE 4:**
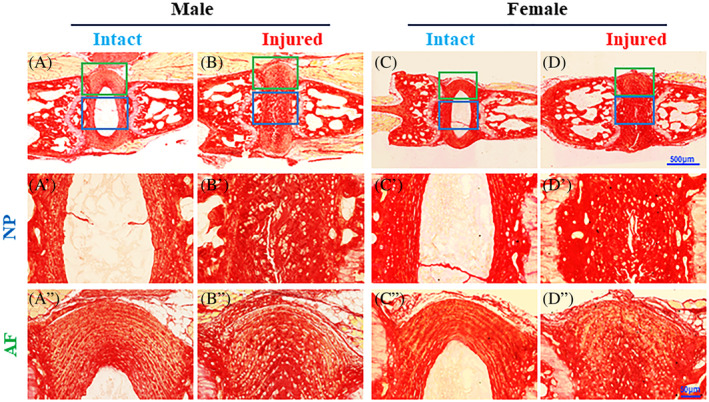
Histological changes in DBA mice 1 week after coccygeal intervertebral disc injury. Intact and adjacent injured IVDs were stained with Picrosirius red; A′‐D′ and A″‐D″ are magnified images in squares in A‐D, respectively. NP, nucleus pulposus; AF, annulus fibrosis. Scale bar in panel D represents 500 μm; scale bar in D" represents 50 μm

We have examined histological features of the mouse tails following injury in mice on the DBA background, with Picrosirius red, to reveal changes in the content and orientation of the collagen bundles. There were consistent histological changes in both male and female IVDs post injury (n = 3‐9; Figure 4). Specifically, normal NP and AF architecture is revealed by Picrosirius red staining, in both the male and female mice (Figure 4A‐A″, C‐C″). Following injury, the IVD tissue showed loss of normal NP architecture, with collagen‐rich scar tissue replacing the normal NP (Figure 4B′,D′). Annular rings were distorted with some interruptions following injury (Figures 1D″ and 4B). Consistent with prior findings,^32^ we did not detect any significant differences in histological features between the male and female mice.

## 4 DISCUSSION

The TNFAIP8 family consists of four members, and recently has been found to regulate inflammatory processes, by interacting with inflammatory pathways and directing lymphocyte migration.^4^, ^14^ TNFAI8 and TIPE2‐deficient mice have been generated,^10^, ^11^ and may prove useful in studying inflammatory processes such as back pain and osteoarthritis in the musculoskeletal field. In the present manuscript, we described gene expression in intact and injured IVDs, which serves as baseline information for future mechanistic studies aimed at explaining potential roles of TNFAIP8 family members in homeostasis of the normal discs and their response to injury.

In the intact IVDs, we were able to separate the NP and AF tissues based on gelatinous texture of the NP and presence of the annular rings in the AF. Gene expression of the four *Tnfaip8* family members was analyzed separately (Figure 1). Similar to the profile of extracellular matrix and adhesion molecule genes,^33^ the TNFAIP8 family of genes tend to be more highly expressed in the AF than in NP. Among the four family members, the *Tnfaip8* gene expression level is the highest, and *Tipe2* gene expression the lowest, with its level barely measurable. This finding suggests that *Tnfaip8* may be expressed by native disc tissues, and possibly important for normal tissue maintenance. *Tipe2*, known to be expressed mainly by leukocytes,^4^ may perform immune functions. This division of labor by molecules of the same family is reminiscent of the two subtypes of cyclooxygenases.^38^

The NP and AF tissues could no longer be reliably separated 1 week following needle puncture (Figure 4). Therefore, the tissues were analyzed as whole IVDs. In response to injury, *Tnfaip8* gene expression decreased while *Tipe2* and *Tipe3* gene expression increased (Figure 2). These findings suggest that TNFAIP8 family members may perform different functions in the injury/repair process. TIPE2, preferentially found in leukocytes, may be expressed by infiltrating macrophages in response to injury, which has been shown in human degenerating IVDs.^20^, ^39^ Confirming or refuting this hypothesis is an important future direction. Future work could include in situ hybridization to show the distribution of mRNAs, and immunostaining to reveal the distribution of TNFAIP8 family protein and leukocyte markers, to identify the cell types that produce these molecules. One limitation of the study is that we have found subtle differences in biomechanical properties between bone‐disc‐bone motion segments of the mouse tail (Zhang‐Mauck, unpublished data), but we have not examined the effects of the mouse tail levels on gene expression. This should be examined in the future.

Inclusion of sex as a biological variable in animal and human studies has been mandated by the NIH recently. This initiative is intended to rectify a prior model where biomedical research focused predominantly on male animals/subjects, potentially obscuring key understanding of sex influences on health processes and outcomes. However, including both sexes in experimental design would increase the animal number needed to design experiments with adequate power for data analysis, in order to yield unbiased and reproducible results. We have recently found and reported subtle differences in extracellular matrix and inflammatory gene expression between male and female mice.^32^ In the current study, we have found that *Tnfaip8* and *Tipe3* gene expression is significantly higher in female control than in their male counterparts (Figure 2A and D), while *Tnfα* gene expression is higher in both intact and injured male discs than in female tissues (Figure 3A). These findings, together with our previous findings,^32^ confirm the need for studies in both male and female animals in the disc injury model.

*Tnfα* gene expression was higher in injured than in intact control IVDs 1 week post injury in both male and female mice (Figure 3A). TNFα may induce TNFAIP8 family members, although simply showing a concurrent gene expression change here does not prove causality. TNFα treatment has been shown to alter F‐actin and alpha‐tubulin in AF cells, suggestive of altered cytoskeletal stiffness.^40^ Anti‐TNFα (infliximab) significantly inhibited pro‐inflammatory cytokine production, while anti‐IL6 did not. TNFα altered AF cell mechanobiology with cytoskeletal remodeling that potentially sensitized AF cells to mechanical strain and increased TNFα‐induced pro‐inflammatory cytokine production.^40^ Exposure to TNFα induced expression of additional pro‐inflammatory cytokines and altered IVD mechanical behavior.^41^ One potential mechanism of TNFα‐induced cytoskeleton change may be by altering the function of the TNFAIP8 family members, since they have been found recently to direct leukocyte migration by reorganizing the cytoskeleton.^14^ Examining leukocyte infiltration in the injured IVDs would be an important future direction, to determine the relative contribution of inflammatory mediators by resident disc cells and infiltrating leukocytes.

TNFα has been shown in herniated and degenerative human IVD tissues.^42^, ^43^, ^44^ The TNFα receptor (TNFR) has two subtypes, TNFR1 and TNFR2, with sometimes opposing functions to maintain the delicate balance of inflammation, cell death, and proliferation.^45^, ^46^ Interestingly, the level of TNFR1 has been positively correlated with back and radicular leg pain, while that of TNFR2 is negatively correlated with pain.^44^ Injecting TNFα into the lumbar IVD induces pain behavior and disc degeneration in rats.^47^ However, some clinical studies have shown efficacy of reducing back and radicular pain by protein factors that regulate TNFα signaling,^48^, ^49^, ^50^ but other such work did not show any benefit.^51^ The inconsistencies in clinical outcomes may be due, in part, to the fact that protein factors require injections, which was delivered either into the epidural space or subcutaneous tissue in the above‐mentioned trials. Permeability into the disc is limited by either method. Furthermore, these reagents block both TNFR1 and TNFR2, and may result in differing effects depending on disease stage, inciting factors, and other individual patient‐related factors such as genetics.

The present work includes a study of IL6, a known marker of IVD degeneration.^20^, ^52^ CXCL1 and ADAM8, subjects of our previous studies,^21^, ^26^, ^31^, ^53^ have also been included. All three genes were found to be elevated in both male and female mice post injury, confirming that they are reliable molecular markers for disc injury. A broader range of molecules warrants examination. For example, we have examined differences in expression of 84 genes between NP and AF.^33^ A similar strategy could be used to examine differences in sex and genetic background in response to injury, in the wild type and TNFAIP8‐deficient mice. Novel methodology, such as RNASeq, could also be utilized to examine an even larger number of genes.

In the aging mouse, the chondrocyte‐like NP cells have been shown to be of notochordal lineage.^54^ Although using lineage‐tracing method is not feasible in humans, it would not be surprising if the human NP cells after age 10 to 12 years of age are also of notochordal origin, although the cell morphology differs from that of notochordal cells in young humans. In addition, all four members of the mammalian TNFAIP8 family are highly conserved in their amino acid sequences.^5^ Further studies in other species are therefore indicated, since it is highly likely that the role of TNFAIP8 family members in local inflammation is translatable to other mammalian species including humans.

In conclusion, genes of the *Tnfaip8* family are expressed in the intact mouse IVDs. *Tipe2* and *Tipe3* genes are elevated in response to injury, along with other known inflammatory markers (ie, *Tnfα, il6, Cxcl1*, and *Adam8*). Surprisingly, the *Tnfaip8* gene was downregulated in response to injury. Future directions include examining tissue distribution of TNFAIP8 family proteins and identifying cells that produce them. Studies examining the mechanisms of actions of this novel family of regulatory proteins in the IVD‐injury model are also indicated. In addition, examining mice that are deficient in TNFAIP8 family of molecules, with respect to gene expression, tissue morphology and mouse behavior, may further delineate the roles of these novel molecules in the etiology of IVD inflammation and degeneration.

## CONFLICT OF INTEREST

The authors declare no financial or other conflict of interest.

## AUTHOR CONTRIBUTIONS

Data acquisition, Zuozhen Tian and Lutian Yao; Research design and/or data interpretation, Honghong Sun, Youhai H. Chen, Ling Qin, Hongtao Zhang, and Yejia Zhang. Drafting and revising manuscript: Yejia Zhang, Honghong Sun, Hongtao Zhang, Ling Qin, and Youhai H. Chen. Statistical analysis: Frances S. Shofer and Yejia Zhang. Storage and access of all primary data: Zuozhen Tian and Yejia Zhang. All authors have read and approved the final version of the manuscript.

## References

[1] Martin BI , Deyo RA , Mirza SK , et al. Expenditures and health status among adults with back and neck problems. JAMA. 2008;299:656‐664. 18270354 10.1001/jama.299.6.656

[2] Weinstein S , Yelin E . United States Bone and Joint Initiative: The Burden of Musculoskeletal Diseases in the United States, Rosemont, IL: United States Bone and Joint Initiative; 2014.

[3] Freundt EC , Bidere N , Lenardo MJ . A different TIPE of immune homeostasis. Cell. 2008;133:401‐402. 18455981 10.1016/j.cell.2008.04.017 PMC2750003

[4] Goldsmith JR , Chen YH . Regulation of inflammation and tumorigenesis by the TIPE family of phospholipid transfer proteins. Cell Mol Immunol. 2017;14:1026. 29176741 10.1038/cmi.2017.127 PMC5719143

[5] Niture S , Dong X , Arthur E , et al. 2018 Oncogenic role of tumor necrosis factor alpha‐induced protein 8 (TNFAIP8). Cells 8: 10.3390/cells8010009. PMC6356598 30586922

[6] Bordoloi D , Banik K , Shabnam B , et al. TIPE family of proteins and its implications in different chronic diseases. Int J Mol Sci. 2018;19:2974 10.3390/ijms19102974. PMC6213092 30274259

[7] Patel S , Wang FH , Whiteside TL , Kasid U . Identification of seven differentially displayed transcripts in human primary and matched metastatic head and neck squamous cell carcinoma cell lines: implications in metastasis and/or radiation response. Oral Oncol. 1997;33:197‐203. 9307729 10.1016/s0964-1955(96)00065-6

[8] Kumar D , Whiteside TL , Kasid U . Identification of a novel tumor necrosis factor‐alpha‐inducible gene, SCC‐S2, containing the consensus sequence of a death effector domain of fas‐associated death domain‐like interleukin‐ 1beta‐converting enzyme‐inhibitory protein. J Biol Chem. 2000;275:2973‐2978. 10644768 10.1074/jbc.275.4.2973

[9] You Z , Ouyang H , Lopatin D , Polver PJ , Wang CY . Nuclear factor‐kappa B‐inducible death effector domain‐containing protein suppresses tumor necrosis factor‐mediated apoptosis by inhibiting caspase‐8 activity. J Biol Chem. 2001;276:26398‐26404. 11346652 10.1074/jbc.M102464200

[10] Sun H , Gong S , Carmody RJ , et al. TIPE2, a negative regulator of innate and adaptive immunity that maintains immune homeostasis. Cell. 2008;133:415‐426. 18455983 10.1016/j.cell.2008.03.026 PMC2398615

[11] Sun H , Lou Y , Porturas T , et al. Exacerbated experimental colitis in TNFAIP8‐deficient mice. J Immunol. 2015;194:5736‐5742. 25948814 10.4049/jimmunol.1401986 PMC4458398

[12] Zhang Z , Liang X , Gao L , et al. TIPE1 induces apoptosis by negatively regulating Rac1 activation in hepatocellular carcinoma cells. Oncogene. 2015;34:2566‐2574. 25043299 10.1038/onc.2014.208

[13] Carmody RJ , Hilliard B , Maguschak K , Chodosh LA , Chen YH . Genomic scale profiling of autoimmune inflammation in the central nervous system: the nervous response to inflammation. J Neuroimmunol. 2002;133:95‐107. 12446012 10.1016/s0165-5728(02)00366-1

[14] Fayngerts SA , Wang Z , Zamani A , et al. Direction of leukocyte polarization and migration by the phosphoinositide‐transfer protein TIPE2. Nat Immunol. 2017;18:1353‐1360. 29058702 10.1038/ni.3866 PMC5690821

[15] Cui J , Hao C , Zhang W , et al. Identical expression profiling of human and murine TIPE3 protein reveals links to its functions. J Histochem Cytochem. 2015;63:206‐216. 25479791 10.1369/0022155414564871 PMC4340736

[16] Fayngerts SA , Wu J , Oxley CL , et al. TIPE3 is the transfer protein of lipid second messengers that promote cancer. Cancer Cell. 2014;26:465‐478. 25242044 10.1016/j.ccr.2014.07.025 PMC4198483

[17] Zhang X , Wang J , Fan C , et al. Crystal structure of TIPE2 provides insights into immune homeostasis. Nat Struct Mol Biol. 2009;16:89‐90. 19079267 10.1038/nsmb.1522

[18] Kim JS , Park J , Kim MS , et al. The Tnfaip8‐PE complex is a novel upstream effector in the anti‐autophagic action of insulin. Sci Rep. 2017;7:6248. 28740220 10.1038/s41598-017-06576-3 PMC5524748

[19] Kepler CK , Markova DZ , Dibra F , et al. Expression and relationship of proinflammatory chemokine RANTES/CCL5 and cytokine IL‐1beta in painful human intervertebral discs. Spine (Phila Pa 1976). 2013;38:873‐880. 23660804 10.1097/BRS.0b013e318285ae08

[20] Shamji MF , Setton LA , Jarvis W , et al. Proinflammatory cytokine expression profile in degenerated and herniated human intervertebral disc tissues. Arthritis Rheum. 2010;62:1974‐1982. 20222111 10.1002/art.27444 PMC2917579

[21] Zhang Y , Chee A , Shi P , et al. Intervertebral disc cells produce interleukins found in patients with back pain. Am J Phys Med Rehabil. 2016;95:407‐415. 26495812 10.1097/PHM.0000000000000399 PMC4841745

[22] Alini M , Eisenstein SM , Ito K , et al. Are animal models useful for studying human disc disorders/degeneration? Eur Spine J. 2008;17:2‐19. 17632738 10.1007/s00586-007-0414-y PMC2365516

[23] Yang F , Leung VY , Luk KD , Chan D , Cheung KM . Injury‐induced sequential transformation of notochordal nucleus pulposus to chondrogenic and fibrocartilaginous phenotype in the mouse. J Pathol. 2009;218:113‐121. 19288580 10.1002/path.2519

[24] Martin JT , Gorth DJ , Beattie EE , Harfe BD , Smith LJ , Elliott DM . Needle puncture injury causes acute and long‐term mechanical deficiency in a mouse model of intervertebral disc degeneration. J Orthop Res. 2013;31:1276‐1282. 23553925 10.1002/jor.22355 PMC6684036

[25] Piazza M , Peck SH , Gullbrand SE , et al. Quantitative MRI correlates with histological grade in a percutaneous needle injury mouse model of disc degeneration. J Orthop Res. 2018;36:2771‐2779. 29687490 10.1002/jor.24028 PMC6200662

[26] Tian Z , Ma X , Yasen M , et al. Intervertebral disc degeneration in a percutaneous mouse tail injury model. Am J Phys Med Rehabil. 2018;97:170‐177. 28863006 10.1097/PHM.0000000000000818 PMC5823709

[27] Pietrosimone KM , Jin M , Poston B , Liu P . Collagen‐induced arthritis: a model for murine autoimmune arthritis. Bio Protoc. 2015;5(20):e1626 10.21769/bioprotoc.1626. PMC4629450 26539560

[28] Zack MD , Arner EC , Anglin CP , Alston JT , Malfait AM , Tortorella MD . Identification of fibronectin neoepitopes present in human osteoarthritic cartilage. Arthritis Rheum. 2006;54:2912‐2922. 16948117 10.1002/art.22045

[29] Zack MD , Malfait AM , Skepner AP , et al. ADAM‐8 isolated from human osteoarthritic chondrocytes cleaves fibronectin at ala(271). Arthritis Rheum. 2009;60:2704‐2713. 19714641 10.1002/art.24753

[30] Zack MD , Melton MA , Stock JL , et al. Reduced incidence and severity of experimental autoimmune arthritis in mice expressing catalytically inactive a disintegrin and metalloproteinase 8 (ADAM8). Clin Exp Immunol. 2009;158:246‐256. 19737139 10.1111/j.1365-2249.2009.04009.x PMC2768814

[31] Ruel N , Markova DZ , Adams SL , et al. Fibronectin fragments and the cleaving enzyme ADAM‐8 in the degenerative human intervertebral disc. Spine (Phila Pa 1976). 2014;39:1274‐1279. 25010013 10.1097/BRS.0000000000000397 PMC4229950

[32] Brent JM , Tian Z , Shofer FS , et al. Influence of genetic background and sex on gene expression in the mouse (*Mus musculus*) tail in a model of intervertebral disc injury. Comp Med. 2020;70:131‐139. 32156324 10.30802/AALAS-CM-19-000034 PMC7137552

[33] Zhang Y , Tian Z , Ashley JW , et al. Extracellular matrix and adhesion molecule gene expression in the normal and injured murine intervertebral disc. Am J Phys Med Rehabil. 2019;98:35‐42. 30085932 10.1097/PHM.0000000000001012 PMC8829805

[34] Ye J , Coulouris G , Zaretskaya I , et al. Primer‐BLAST: a tool to design target‐specific primers for polymerase chain reaction. BMC Bioinformatics. 2012;13(1):134. 22708584 10.1186/1471-2105-13-134 PMC3412702

[35] Livak KJ , Schmittgen TD . Analysis of relative gene expression data using real‐time quantitative PCR and the 2(−delta delta C[T]) method. Methods. 2001;25:402‐408. 11846609 10.1006/meth.2001.1262

[36] Schmittgen TD , Livak KJ . Analyzing real‐time PCR data by the comparative C(T) method. Nat Protoc. 2008;3:1101‐1108. 18546601 10.1038/nprot.2008.73

[37] SAS institute inc . 2002–2004. SAS 9.1.3; help and documentation. cary, NC.

[38] Fitzpatrick FA . Cyclooxygenase enzymes: regulation and function. Curr Pharm Des. 2004;10:577‐588. 14965321 10.2174/1381612043453144

[39] Nakazawa KR , Walter BA , Laudier DM , et al. Accumulation and localization of macrophage phenotypes with human intervertebral disc degeneration. Spine J. 2018;18:343‐356. 29031872 10.1016/j.spinee.2017.09.018 PMC5815908

[40] Likhitpanichkul M , Torre OM , Gruen J , Walter BA , Hecht AC , Iatridis JC . Do mechanical strain and TNF‐alpha interact to amplify pro‐inflammatory cytokine production in human annulus fibrosus cells? J Biomech. 2016;49:1214‐1220. 26924657 10.1016/j.jbiomech.2016.02.029 PMC4913356

[41] Walter BA , Likhitpanichkul M , Illien‐Junger S , Roughley PJ , Hecht AC , Iatridis JC . TNFalpha transport induced by dynamic loading alters biomechanics of intact intervertebral discs. PLoS ONE. 2015;10:e0118358. 25734788 10.1371/journal.pone.0118358 PMC4348425

[42] Ohtori S , Inoue G , Eguchi Y , et al. Tumor necrosis factor‐alpha‐immunoreactive cells in nucleus pulposus in adolescent patients with lumbar disc herniation. Spine (Phila Pa 1976). 2013;38:459‐462. 22990365 10.1097/BRS.0b013e3182739cb4

[43] Le Maitre CL , Hoyland JA , Freemont AJ . Catabolic cytokine expression in degenerate and herniated human intervertebral discs: IL‐1beta and TNFalpha expression profile. Arthritis Res Ther. 2007;9:R77. 17688691 10.1186/ar2275 PMC2206382

[44] Andrade P , Visser‐Vandewalle V , Philippens M , et al. Tumor necrosis factor‐alpha levels correlate with postoperative pain severity in lumbar disc hernia patients: opposite clinical effects between tumor necrosis factor receptor 1 and 2. Pain. 2011;152:2645‐2652. 21920667 10.1016/j.pain.2011.08.012

[45] Wajant H , Siegmund D . TNFR1 and TNFR2 in the control of the life and death balance of macrophages. Front Cell Dev Biol. 2019;7:91. 31192209 10.3389/fcell.2019.00091 PMC6548990

[46] Yang S , Xie C , Chen Y , et al. Differential roles of TNFalpha‐TNFR1 and TNFalpha‐TNFR2 in the differentiation and function of CD4(+)Foxp3(+) induced treg cells in vitro and in vivo periphery in autoimmune diseases. Cell Death Dis. 2019;10:27‐018‐1266‐6. 30631042 10.1038/s41419-018-1266-6 PMC6328545

[47] Lai A , Moon A , Purmessur D , et al. Annular puncture with tumor necrosis factor‐alpha injection enhances painful behavior with disc degeneration in vivo. Spine J. 2016;16:420‐431. 26610672 10.1016/j.spinee.2015.11.019 PMC4913353

[48] Ohtori S , Miyagi M , Eguchi Y , et al. Epidural administration of spinal nerves with the tumor necrosis factor‐alpha inhibitor, etanercept, compared with dexamethasone for treatment of sciatica in patients with lumbar spinal stenosis: a prospective randomized study. Spine (Phila Pa 1976). 2012;37:439‐444. 22020607 10.1097/BRS.0b013e318238af83

[49] Freeman BJ , Ludbrook GL , Hall S , et al. Randomized, double‐blind, placebo‐controlled, trial of transforaminal epidural etanercept for the treatment of symptomatic lumbar disc herniation. Spine (Phila Pa 1976). 2013;38:1986‐1994. 24165696 10.1097/01.brs.0000435140.61593.4c

[50] Genevay S , Viatte S , Finckh A , Zufferey P , Balagué F , Gabay C . Adalimumab in severe and acute sciatica: a multicenter, randomized, double‐blind, placebo‐controlled trial. Arthritis Rheum. 2010;62:2339‐2346. 20506391 10.1002/art.27499

[51] Okoro T , Tafazal SI , Longworth S , Sell PJ . Tumor necrosis alpha‐blocking agent (etanercept): a triple blind randomized controlled trial of its use in treatment of sciatica. J Spinal Disord Tech. 2010;23:74‐77. 20072036 10.1097/BSD.0b013e31819afdc4

[52] Weber KT , Alipui DO , Sison CP , et al. Serum levels of the proinflammatory cytokine interleukin‐6 vary based on diagnoses in individuals with lumbar intervertebral disc diseases. Arthritis Res Ther. 2016;18:3 10.1186/s13075-015-0887-8. 26743937 PMC4718017

[53] Zhang Y , Chee A , Shi P , et al. Allogeneic articular chondrocyte transplantation downregulates interleukin 8 gene expression in the degenerating rabbit intervertebral disk in vivo. Am J Phys Med Rehabil. 2015;94:530‐538. 25133623 10.1097/PHM.0000000000000194 PMC4329109

[54] Mohanty S , Pinelli R , Pricop P , Albert TJ , Dahia CL . Chondrocyte‐like nested cells in the aged intervertebral disc are late‐stage nucleus pulposus cells. Aging Cell. 2019;18:e13006. 31290579 10.1111/acel.13006 PMC6718620

